# Regime for Bowel Preparation in Patients Scheduled to Colonoscopy: Low-Residue Diet or Clear Liquid Diet? Evidence From Systematic Review With Power Analysis

**DOI:** 10.1097/MD.0000000000002432

**Published:** 2016-01-08

**Authors:** Guo-Min Song, Xu Tian, Li Ma, Li-Juan Yi, Ting Shuai, Zi Zeng, Xian-Tao Zeng

**Affiliations:** From the Department of Nursing, Tianjin Hospital, Tianjin, China (G-MS); Graduated College, Tianjin University of Traditional Chinese Medicine, Tianjin, China (XT, LM, L-JY, TS, ZZ); and Center for Evidence-Based and Translational Medicine, Zhongnan Hospital of Wuhan University, Wuhan, China (XT, ZZ).

## Abstract

Clear liquid diet (CLD) is used to perform bowel preparation before colonoscopy traditionally, but several clinical studies indicated that low-residue diet (LRD) generates equal effects to CLD and a conclusive conclusion has not yet been yielded. The systematic review was performed to address this conflict and facilitate informed decision-making eventually.

To capture randomized controlled trials (RCTs) comparing LRD with CLD in terms of bowel preparation, a search was performed in PubMed, EMBASE, Cochrane Central Register of Controlled Trials (CENTRAL), Science Direct, recent conference abstracts, Google Scholar, and Clinicaltrials.gov through May 2015. We performed all meta-analyses based on fixed- or random-effects model, which is generated from clinical characteristics and methodology. Moreover, the G^∗^Power software was adopted to achieve statistical power for each outcome.

In total, we captured 109 potential citations at initial search stage and 2 topic-related articles were included through other sources. After critical appraisal, 7 RCTs were eligible for our inclusion criteria. Meta-analyses generated similar effects in bowel preparation quality, efficacy of colon cleansing, and compliance with recommended dietary regime when LRD versus CLD regime, but patients who were prescribed to receive LRD have slightly better tolerance (RR, 1.06; 95% CI, 1.02–1.11) and tended to repeat the same preparation regime in future (RR, 1.17; 95% CI, 1.09–1.26) relative to patients in CLD. Importantly, both regimes resulted in similar adverse events (AEs).

With the best available evidence, LRD could be recommended to be as standard regime for bowel preparation prior to colonoscopy.

## Abstract 2

Supplemental Digital Content is available in the text

## INTRODUCTION

Despite some advancement in prevention, diagnosis, and treatment of malignancy over last decades, colorectal cancer (CRC) is still the third contributor to the cancer-death in United States.^1^ Colonoscopy is an effective approach to detect and remove the precancerous lesions^2^ and issued data well demonstrate that successful implementation of colonoscopy can reduce ∼50% mortality rate of CRC.^3,4^ Consequently, the colonoscopy technique has been recommended to screen and treat CRC.^2^ Significantly, however, adequate bowel preparation plays a key role in successful implementation of colonoscopy and is the guarantee of proper screening efficacy.^5^ Published studies revealed that ∼20% to 25% inadequate bowel preparations were caused mainly by low patient-based compliance, poor palatability of bowel preparation solution, and large volume preparation solution.^6,7^ It should be noted that, however, low patient-based compliance is the critical one in numerous reasons of causing inadequate bowel preparation.^8,9^

Clear liquid diet (CLD) is an established modified regime of normal diet, which do not include any solids, milk, and fruit juices containing pulp.^10^ Traditionally, CLD is extensively adopted to perform bowel preparation on the day prior to colonoscopy.^5^ However, these shortages of the bowel preparation regime, such as too restrictive, containing insufficient calories and high risk of causing several adverse events (AEs), significantly impair the patient-based compliance.^11,12^

To address these issues existed in CLD regime, researchers and clinical practitioners explored some novel bowel preparation regimes, of which low-residue diet (LRD) regime is increasingly concerned. LRD is also a modified regime of normal diet, which contain daily <10 to 15 g fiber.^13^ Several published randomized controlled trials (RCTs) have investigated the comparative efficacy of LRD relative to CLD in improving the quality of bowel preparation in patients scheduled to colonoscopy;^5,11,12,14,15^ however, the conclusion remains unclear. Nguyen and colleagues performed a study with meta-analysis to systematically assess the efficacy of LRD compared to CLD in implementing bowel preparation.^16^ These authors incorporated 5 eligible RCTs including 870 patients into their study. The satisfaction with bowel preparation, patient-based tolerance, patient-based willing to repeat the same preparation regime in future, and AEs were listed to be as the outcomes of interesting in this study. It must be noted that, however, the insufficient recall ratio may decrease the power of their study. Moreover, their study did not include the primary outcomes for bowel preparation on the day prior to colonoscopy, such as the quality of bowel preparation and the efficacy of colon cleansing.

Conflict conclusions will confound the informed decision-making and obstacle clinical practice eventually. Consequently, we performed this systematic review and meta-analysis of RCTs concerning comparative efficacy of LRD relative to CLD in performing the bowel preparation on the day prior to colonoscopy to facilitate evidence-based practice.

## METHODS

The Preferred Reporting Items for Systematic Review and Meta-analysis (PRISMA),^17^ Cochrane Handbook for Systematic Reviews of Interventions Version 5.1.0^18^ and guideline issued by Center for Review and Dissemination (CRD)^19^ were compiled to improve the reporting quality of our study. We also registered the prospective protocol of this systematic review and meta-analysis on PROSPERO database and a registration number of CRD42015023929 has been approved (http://www.crd.york.ac.uk/prospero/). Ethical approval and patient informed consent were not needed because all analyses were performed on the basis of previous information.

### Selection Criteria

We prespecified study selection criteria according to the PICOS acronym (population, intervention, comparison, and outcome), and the details are as follows: (1) *P*: all adults with age ≥18 years scheduled for colonoscopy were eligible for our study. (2) *I* and *C:* LRD versus CLD. (3) *O*: the quality of bowel preparation (excellent–good preparation), efficacy of colon cleansing, patient tolerance, willing to repeat, compliance with dietary regime, and adverse events (AEs) will be evaluated in our study. (4) *S*: only RCTs that comparing LRD with CLD in terms for bowel preparation prior to colonoscopy were included into our study. Any divergences between authors concerning the eligibility of a study were resolved by consulting a third author until a consensus was reached.

### Literature Search

Comprehensive literature search is the core to guarantee generation of reliable and valid meta-analysis results. Two investigators were assigned to independently search potential studies comparing LRD with CLD in terms of bowel preparation before colonoscopy in PubMed, EMBASE, Science Direct, the Cochrane Central Register of Controlled Trials (CENTRAL), recent abstracts from major conference proceedings (Digestive Diseases Week, American College of Gastroenterology), Google Scholar, and Clinicaltrials. gov (http://clinicaltrials.gov) through May 2015. The search terms used to perform the search process were as following: “colonoscop^∗^,” “Colonoscopic Surgical Procedure^∗^,” “Colonoscopic Surger^∗^,” “low-residue diet^∗^,” “low residue diet^∗^,” LRD, NutraPrep, “low residue product^∗^,” “low-residue product^∗^,” “clear liquid diet^∗^,” “clear-liquid diet^∗^,” CLD, “clear liquid product^∗^,” “clear-liquid product^∗^,” “clear water,” random^∗^. We summarized all search strategies in Additional File 1. We also manually searched the reference lists of topic-related reviews and eligible studies to include any latent articles and eventually guarantee recall ratio. Only studies in English were included. Consensus is the approach to address the divergences concerning the search of citations and eligibility of information.

### Study Selection and Data Extraction

After obtaining eligible full text, 2 independent investigators to abstract the information using predesigned standard data extraction form (Additional File 2). If essential information for our analysis was inadequate, we will contact associated author. Any disagreements occurred at this stage were resolved by consulting a third investigator.

We prespecified all outcomes of interest prior to conducting this full-text systematic review and meta-analysis. The primary consists of the quality of bowel preparation and efficacy of colon cleansing. For these primary indices, Boston Bowel Preparation Scale (BBPS), Ottawa Bowel Preparation Scale (OBPS), Aronchik scale or other rating scales were selected to evaluate the efficacy of bowel preparation in accordance with specific criteria. Patient tolerance to bowel preparation regime, willings to repeat the same bowel preparation regime in future and adverse events (AEs) were specified as secondary outcome measures of interest in this study. These indices were assessed by self-reported or recorded by using nonstandardized questionnaires administered after performing the bowel preparation, and all data for secondary outcomes were extracted from associated questionnaires.

### Assessing the Risk of Bias and Grading the Quality of Evidence

We adopted the Cochrane risk of bias assessment instrument to assess the methodology quality of each of eligible study.^18,20^ Six domains including selection bias, performance bias, detection bias, attrition bias, reporting bias, and other bias were critically assessed accordingly for the purpose of judging the level of risk of bias of each included study. Each domain was rated as “high bias risk,” “unclear bias risk” or “low bias risk” depends on the match degree between information extracted and assessment criteria.^18^ A point should be noted is that study which explore the adequacy of bowel preparation cannot be logistically designed as double blinding, the performance bias domain was rated as “low bias risk” if the endoscopist was blinded to the protocol and other medical staffs and patients were instructed not to discuss the protocol-related information with the given endoscopist.

We used the Grades of Recommendation, Assessment, Development and Evaluation system (GRADE) to grade the qualities of evidence for all outcomes. The level of evidence will be graded as high, moderate, low or very low on the basis of assessing risk of bias, inconsistent, indirectness, imprecision, and other considerations.^21^ We used the GRADE profiler software version 3.6 to evaluate the quality of evidences (http://www.gradeworkinggroup.org/).

### Statistical Analysis

All analyses were performed using RevMan 5.3 (Copenhagen: The Nordic Cochrane Centre, The Cochrane Collaboration, 2013). The pooled effect was expressed as relative risk (RR) and the standard mean difference (SMD) with 95% confidence intervals (CIs) for dichotomous and continuous data, respectively. We also qualitatively evaluated heterogeneity across studies using Cochrane's Q statistic with *P* value. The degree of heterogeneity was quantified using *I*^*2*^ statistic, which is defined as the variation degree can be explained by heterogeneity. An *I*^*2*^ of ≥50% or *P* < 0.1 indicated that pooled estimates may be impaired by heterogeneity. In contrast, if *I*^*2*^ was <50% and *P* > 0.1, the studies were considered to be homogeneous. Each separate meta-analysis was performed via a random-model based on Mantel-Haenszel (MH) or a fixed-effects model based on the inverse variance (IV) statistical approach according to the clinical characteristic and methodology of eligible studies pooled. Subgroup analysis was conducted according to various measurements.^22^ A qualitative analysis was used to describe the studies, in which data was incomplete, heterogeneity can affect the pooled results or lack of a number of studies to pool. Owing to the limited number (below 10) of studies included in each analysis, publication bias was not assessed.^23^ Moreover, we conducted power analyses of eligible individual studies and meta-analyses using G^∗^Power software (version 3.1.9.2).^24^

## RESULTS

### Identification and Selection of RCTs

**FIGURE 1:**
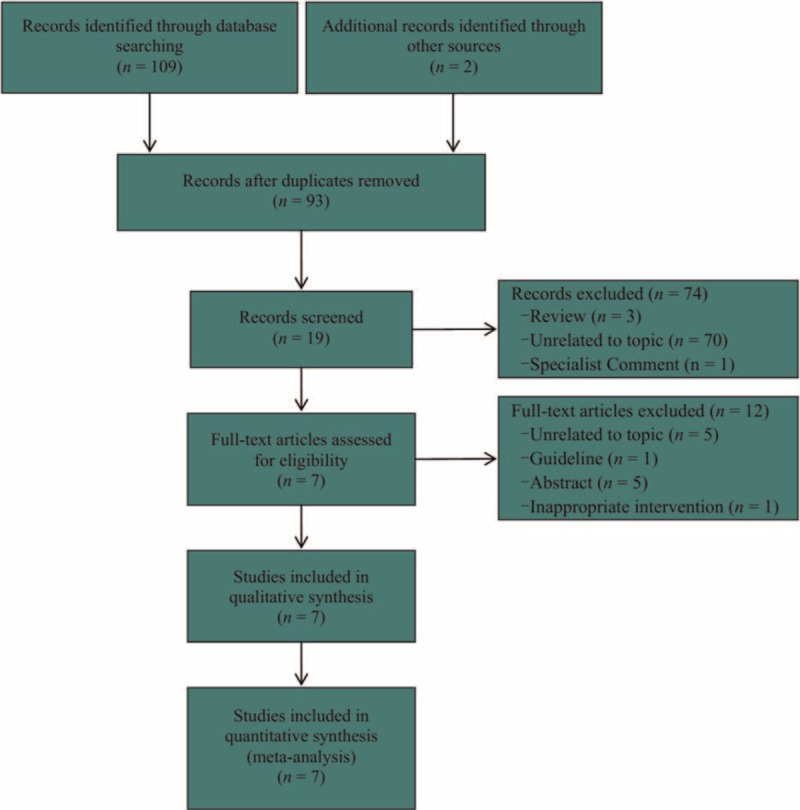
Flow diagram of literature retrieval and selection: 109 potential citations and additional 2 records were initially obtained and eventually 7 eligible studies were incorporated into this systematic review and meta-analysis.

We captured 109 citations at the initial search stage. The EndNote version 7.1 was used to sort all records. Eighteen were eliminated from all due to duplicate, and 74 were classified into exclusion file after screening title and abstract. Of which, 3, 1, and 70 were review, specialist comment, and unrelated to our topic studies, respectively. We further assessed the full-text of remaining records, and 12 studies were excluded due to unrelated to our topic, guideline, abstract file, and inappropriate target colonoscopy technique (patients scheduled to accept the PillCam Colon Capsule Endoscopy). As a result, we incorporated 7 eligible studies^5,11,12,14,25–27^ into our study ultimately. Of these 7 articles, 2^25,27^ were included based on checked references of eligible and function of related citations of electronic database. Figure 1 displayed the process of retrieval and selection of RCTs.

### Basic Information of All Eligible RCTs

Table 1 presented the basic information of all eligible RCTs. The publication year of all eligible studies spanned from 2005 to 2014. For all eligible studies, 5 were performed in USA. One study^5^ conducted by Rapier and colleagues was a 3-arm design, and thus we only extracted corresponding information that was applicable to our topic. Consequently, sample size of each study varied from 75 to 506 and the sum was 1590. Three studies^11,14,26^ reported the number of patients experienced the colonoscopy previously. The quality of bowel preparation was judged by using criteria of “poor,” “intermediate,” “good,” and “excellent” in study performed by Delegge et al^14^ and Scott et al,^27^ Melicharkova et al^25^ and Park et al^26^ used OBPS to assess the adequacy of bowel preparation, Rapier et al^5^ assessed this given outcome of interest using 5 point self-made scale of “excellent,” “good,” “fair,” “poor,” and “very poor,” and Sipe et al^12^ and Stolpman et al^11^ adopted BBPS to evaluate this index. Corresponding custom questionnaires and self-reported methods were used to measure the status of other outcomes.

**TABLE 1:**
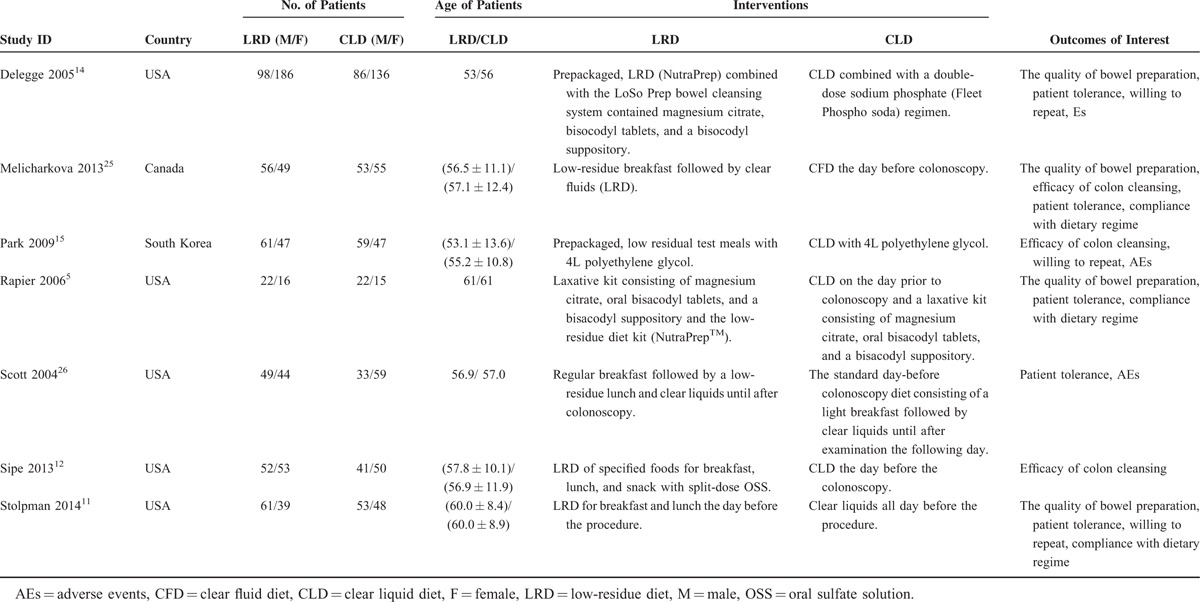
Basic Characteristics of 7 Trials Included into this Systematic Review and Meta-Analysis

### Methodological Quality of Eligible RCTs

**FIGURE 2:**
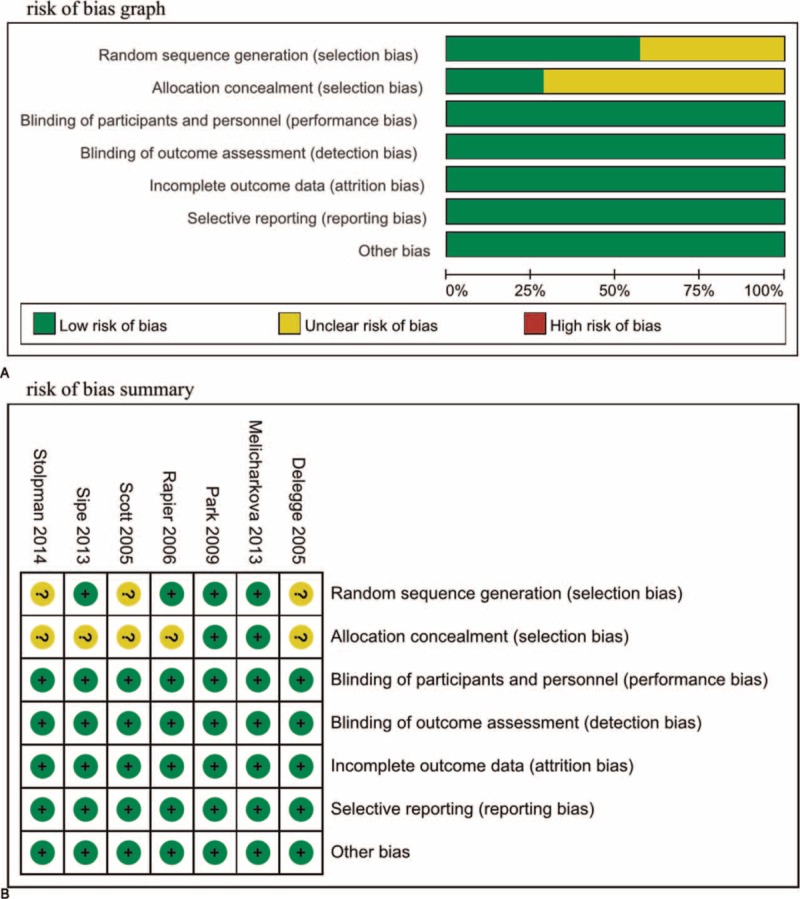
Assessment of risk of bias: (A) risk of bias graph, (B) risk of bias summary; the whole quality of all eligible studies was good because most of risk of bias indices were rated as low risk and no index was graded as high risk.

We evaluated the risk of bias of each eligible study using the Cochrane Risk of Bias Tool. Of all eligible studies, 4 generate appropriately random sequence using random number table,^25^ stratified randomization,^26^ and computer-generated randomization table.^5,12^ Two eligible adopted sealed opaque envelope^25^ and specific coordinator^26^ to carried out allocation of randomization. All studies performed blinding to colonoscopists. All studies reported the number and reasons of drop-out and Melicharkova and colleagues^25^ adopted intention–to–treat (ITT) to analyze results. All eligible studies reported the results of expected outcomes of interest. Figure 2 graphically presented the methodological quality.

### Quality of Evidence

We reported the quality of evidence for each outcome except for individual AEs in the Table 2. The quality of bowel preparation (excellent–good preparation), efficacy of colon cleansing and overall AEs were listed as critical outcome and remaining outcomes were important. For the efficacy of colon cleansing, we provided separate quality of evidence based on subgroup, which was conducted according to different measurements. The levels of evidence for the quality of bowel preparation (excellent–good preparation), patient tolerance, willings to repeat the same diet regime in future, and overall AEs were moderate. The level of evidence for efficacy of colon cleansing and compliance with dietary regime were rated as low and very low, respectively.

**TABLE 2:**
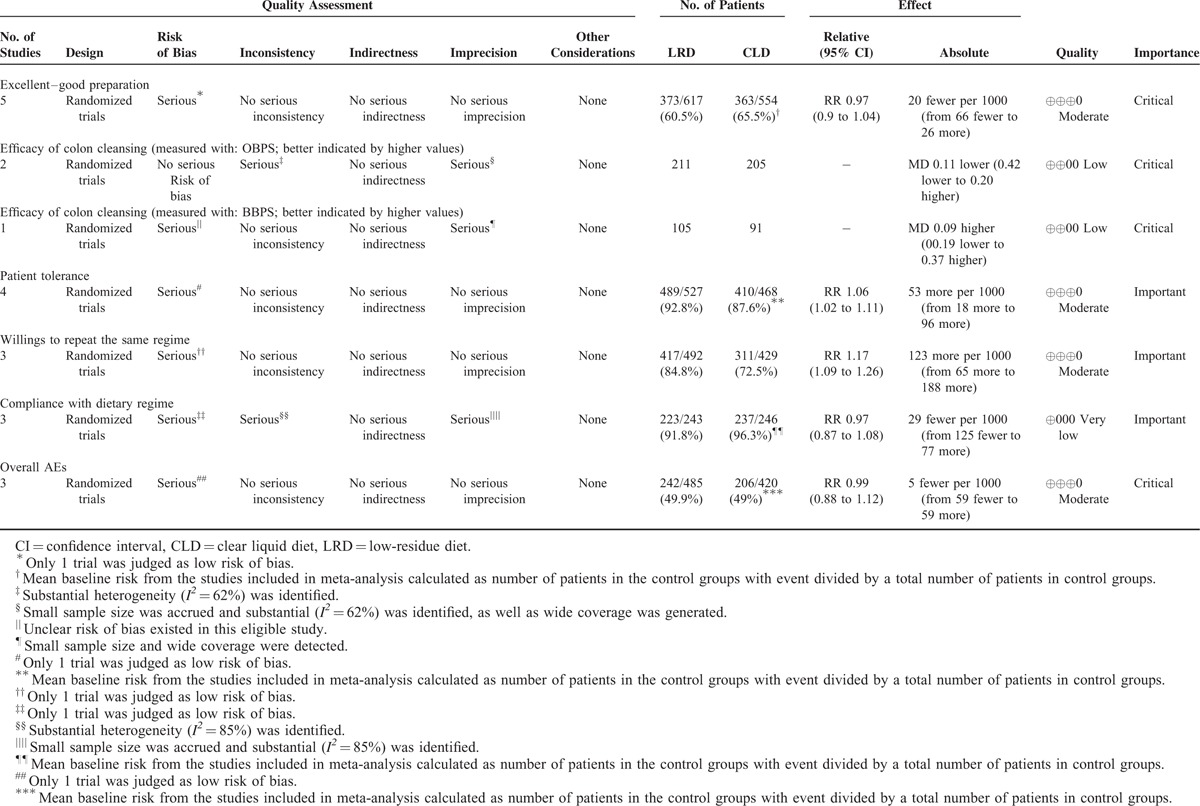
GRADE Evidence Profile

### The Quality of Bowel Preparation

**FIGURE 3:**
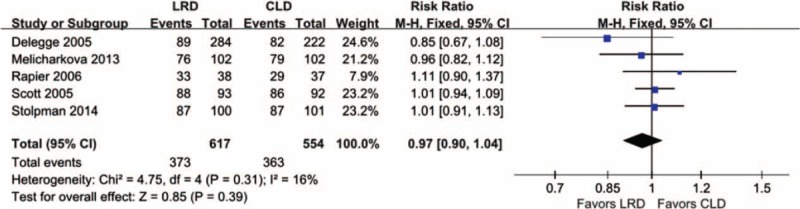
Meta-analysis on the quality of bowel preparation (excellent—good preparation): 5 eligible studies including 1171 participants were included and no significant difference for this given outcome was identified based on a fixed-effect model.

Of all eligible studies, 5^5,11,14,25,27^ including 1171 participants reported the excellent or good bowel preparation and were incorporated into this meta-analysis. We adopted the fixed-effects model to compute the summary effect estimate because no clinical characteristics and methodological differences were detected in these 5 studies, and the heterogeneity (*I*^2^ = 16%, *P* = 0.31) was at the level supported to select this model. This meta-analysis indicated no difference between dietary regimes in terms of excellent or good bowel preparation (RR, 1.01; 95% CI, 0.91–1.13; *P* = 0.39), and the forest plot was presented in Figure 3. The pooled result on this given outcome of interest was supported by moderate level evidence.

### Efficacy of Colon Cleansing

**FIGURE 4:**
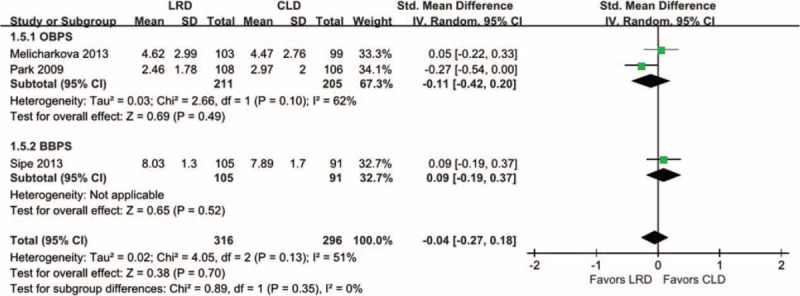
Meta-analysis on the efficacy of colon cleansing: subgroup analyses according to OBPS and BBPS were not statistically significant. BBPS = Boston Bowel Preparation Scale, OBPS = Ottawa Bowel Preparation Scale.

Three eligible studies,^12,25,26^ in which 316 and 296 participants were randomly divided into LRD and CLD arm, respectively, were included into this separate meta-analysis because data on efficacy of colon cleansing were extracted from them. Two studies^25,26^ used OBPS to evaluate this index and remaining^12^ adopted BBPS to do it. Consequently, a subgroup analysis was conducted based on different measurements. No difference was identified between regimes in terms of efficacy of colon cleansing based on random-effects model subgroup analysis, which was supported by low level evidence (SMD, −0.04; −0.27 to 0.18; *P* = 0.70). Figure 4 presented the graphical results.

### Patient Tolerance

**FIGURE 5:**
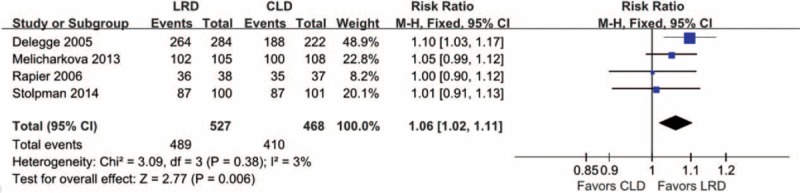
Meta-analysis on patient tolerance to the same diet regime in future: 4 eligible studies which enrolled 995 participants reported this given outcome and patients in LRD diet regime group reported better tolerance based on a random-effect model. LRD = low-residue diet.

A total of 7 studies were incorporated into our study. Of which 5^5,11,14,25,26^ published the data of patient tolerance to recommended dietary regimes, and 1078 participants were enrolled. However, reporting format on this given outcome in study performed by Park and colleagues^26^ is different from others, and thus only 4 studies were incorporated. The heterogeneity was identified (*I*^*2*^ = 3%, *P* = 0.38), and thus we performed fixed-effects model separate meta-analysis to calculate the summary effect estimate. The moderate level evidence suggested that there was a near-significant difference with more patients reporting tolerance in the LRD group (RR, 1.06; 95% CI, 1.02–1.11; *P* = 0.00). Certainly, the result reported by Park et al also supported that LRD improved the patient tolerance (*P* = 0.03). We also presented corresponding separate and pooled results in Figure 5.

### Willings to Repeat

**FIGURE 6:**
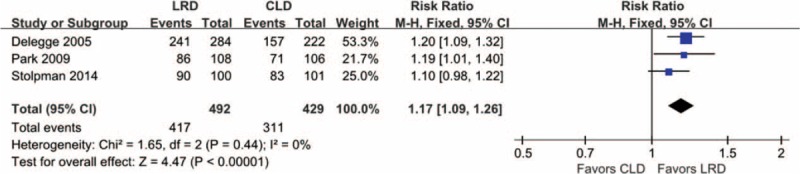
Meta-analysis on willings to repeat the same diet regime: 3 studies including 921 eligible participants were incorporated and the patients were prescribed to use the LRD diet regime; they were allowed to select the same diet regime if a bowel preparation is needed in future according to the synthesis analysis based on a fixed-effect model. LRD = low-residue diet.

Effective information on willings to repeat the same bowel preparation regimes in future was abstracted from 3 eligible studies,^11,14,26^ in which 492 and 429 participants were randomly recruited into LRD and CLD arm, respectively. The heterogeneity test did not detect significant statistical heterogeneity (*I*^*2*^ = 0%, *P* = 0.44); therefore, a fixed-effects model was used to conduct this separate meta-analysis. The meta-analysis indicated that the willings to repeat in LRD arm was higher than that of CLD arm (RR, 1.17; 95% CI, 1.09–1.26; *P* = 0.00). The graphical result can be obtained from Figure 6. Moderate level evidence supported this pooled result on the willings to repeat the same diet regime in future.

### Compliance With Dietary Regime

**FIGURE 7:**
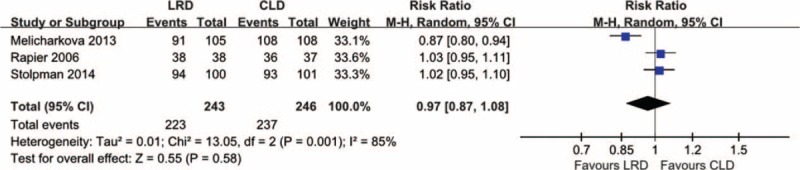
Meta-analysis on compliance with recommended diet regime: 3 eligible studies in which 489 participants were recruited provided the essential information on this outcome and the pooled result based on a random-effect model indicated no statistically significant difference.

We identified 3 eligible studies^5,11,25^ including 489 participants to calculate the pooled effect estimates on compliance with dietary regime. The statistical heterogeneity was detected (*I*^*2*^ = 85%, *P* = 0.00); thus we adopted this random-effects model to perform this separate meta-analysis on compliance with dietary regime. The result of which the compliance in the LRD group was not superior to that of the CLD group was obtained (RR, 0.97; 95% CI, 0.87–1.08; *P* = 0.58) and the forest plot was presented in Figure 7. But it is noted that this result was only supported by very low evidence.

### Adverse Events

**FIGURE 8:**
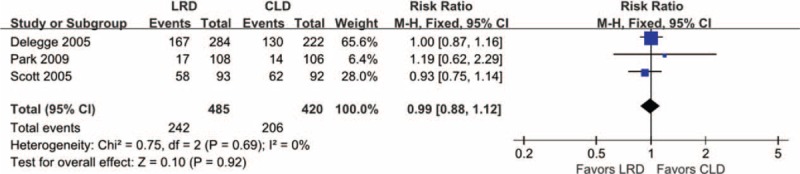
Meta-analysis on overall AEs: 3 studies including 905 were eligible for the inclusion criteria prespecified in our study and the synthesis analysis with fixed-effect model did not indicate statistically significant difference.

Three studies^14,26,27^ included in this systematic review and meta-analysis reported overall adverse events (AEs) and all incorporated to compute the summary effect estimate. We adopted *I*^*2*^ statistic to test heterogeneity across studies and no statistical heterogeneity was identified (*I*^*2*^ = 0%, *P* = 0.69). Consequently, a fixed-effects model was used to conduct this separate meta-analysis. The incidence of AEs in the LRD group was comparable with that of the CLD group (RR, 0.99; 95% CI, 0.88–1.12; *P* = 0.92). The forest plot on overall incidence of AEs was presented in Figure 8.

We performed 6 meta-analyses to separately calculate the summary effect estimates of the most common individual AEs including hunger, bloating, abdominal pain, nausea, vomiting, and headache. These separate meta-analyses results did not detect significant differences between dietary regimes in terms of incidence of individual AEs. Corresponding forest plots were summarized in Table 3.

**TABLE 3:**
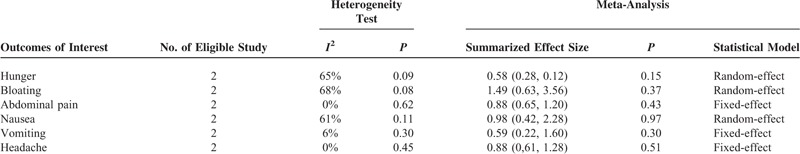
Pooled Results of Individual AEs

### Power Analysis

We adopted the statistical power analysis to reassess the available data when an alpha of 0.05 and beta of 0.2 were assigned. The power of eligible individual study ranged from 0.1% to 99.9%. The powers of most of outcomes in all eligible studies were <50%, which is the level of power to detect a moderate effect size; however, it is noted that the power of patient tolerance and willings to repeat the same diet regime in future in Delegge et al^14^ and willings to repeat preparation in Melicharkova et al^25^ were 82.9%, 96.6%, and 99.9%, respectively. For these 6 outcomes, corresponding power of pooled effect estimates ranged from 5.3% to 99.5%. The specific values of power were summarized in Table 4.

**TABLE 4:**
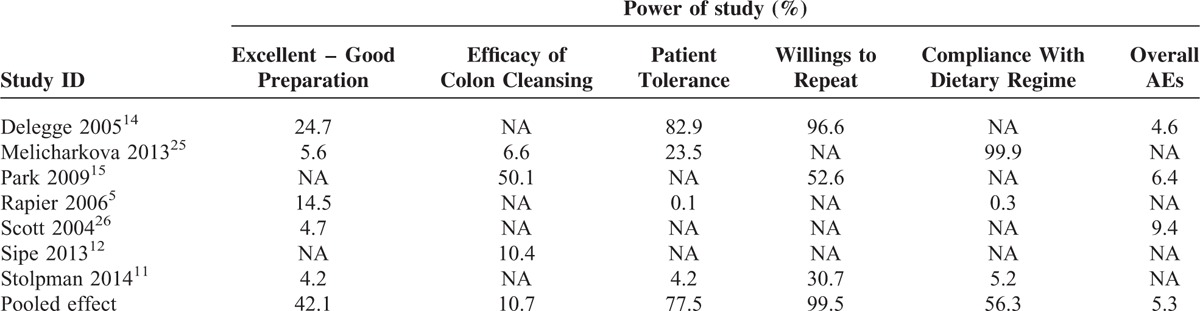
Power Analysis of Eligible Studies and Meta-Analyses

## DISCUSSION

Adequacy of bowel preparation is critical to guarantee the accuracy and proper visualization of colonoscopy test and treatment.^28^ Consequently, several studies were performed to explore the optimal bowel preparation regime prior to colonoscopy. Traditionally, the CLD dietary regime was widely selected to perform the bowel preparation; however, obvious limits such as time-consuming, uncomfortable feelings of the patient, and lack of convenient obstacle the use of this approach.^6^ Although some studies published previously suggested that LRD was superior to CLD in terms of bowel preparation, others indicated no difference between 2 regimes. We performed this systematic review and meta-analysis of RCTs that compared LRD with CLD in terms of bowel preparation prior to colonoscopy to determine whether LRD is not-inferior to the CLD diet regime in terms of bowel preparation. Seven eligible studies with low or unclear risk of bias were eligible for our inclusion criteria based on a comprehensive search and critical appraisal.

Our findings suggested that LRD did not compromise the quality of bowel preparation relative to CLD and it was supported by moderate level evidence. This is consistent with results of most studies included into this separate meta-analysis on the given outcome. As well known, meta-analysis is a statistical method which is used to synthesize the information on the same topic from the homogeneous studies. Consequently, the more sample size will be accrued by using this method. And thus, we generated more accurate effect estimate based on accumulated sample size, and thus decreased the possibility of type 2 error due to more sample size was accumulated. In addition, Spada and colleagues planned a prospective, randomized-controlled trial to investigate the comparative efficacy of LRD compared to CLD before PillCam colon capsule endoscopy and corresponding result also supported our findings. It is noted that this pooled result was consistent with that of conference abstract of meta-analysis previously published.^16^

For efficacy of colon cleansing, our finding supported that these 2 regimes have similar efficacy, and is consistent with most of eligible studies. However, this pooled result on this outcome of interest generated from low level evidence, and thus the power of this outcome should be further explored. Delegge and colleagues^14^ performed study to determine whether a meal could be consumed during standard bowel preparation and obtained a result contrary to our result. Use of cathartic regime and more female participants may be key factors caused this difference. Moreover, a prospective study comparing oral sodium phosphate solution to a bowel cleansing preparation with nutrition food package in children conducted by El-Baba and colleagues supported LRD superior to CLD in terms of colon cleansing; however, sample participates from different population resulted in substantial variation of effects.

Patient tolerance, compliance with dietary regime, and willings to repeat the same bowel preparation regime in future were also evaluated. Our pooled results from moderate level evidence revealed that patients in the LRD regime were more tolerant to recommended bowel preparation regime and tend to select the same regime in future if colonoscopy is necessary. These 2 pooled results were consistent with that of all eligible studies. Not surprisingly, LRD was superior to CLD to improve these 2 outcomes. CLD is beneficial in helping to clear the bowels of residual contents; however, it is often difficult for the patient to not eat any solid food for the long period of time required for bowel cleansing^5^ and these limits could be addressed by using LRD. However, compliance with recommended dietary regime indicated no difference between groups. It is important that the pooled result on compliance with diet regime generated from very low level evidence and thus the comparative effects of LRD versus CLD in terms of compliance with recommend diet regime should be tested through planning further topic-related studies. Although LRD addressed some shortcomings of CLD, the dietary listed in the LRD regime was common difficultly obtained by patients and thus impaired the compliance of them with recommended regime.^5^

In order to evaluate the safety of LRD relative to CLD, we performed meta-analysis to assess the incidence of AEs between groups. First, we analyzed the overall incidence of AEs, the result which was supported by moderate level evidence indicated no significant difference. Second, we conducted 6 separate meta-analyses to assess the incidence of individual AE; these summary results indicted also no difference. Comprehensive analysis on AEs confirmed that the safety of LRD was comparable to that of CLD. However, the statistical power of pooled result on overall AEs is just 5.3%, which is far away from 80%, and thus further RCTs with large scale and high quality are warranted to determine these AEs.

Although only RCT with full-text was fallen into our inclusion criteria, 4 topic-related conference abstracts with sufficient information were worth to further analyses. Butt and colleagues^29^ recruited 226 participants with average age of 53 years to investigate the comparative effectiveness of white diet (similar to LRD) versus CLD and obtained expected results that were consistent with findings in our systematic review and meta-analysis. This study suggested that white diet is not inferior to CLD for the quality of bowel preparation; moreover, patient tolerance and willings to repeat this given regime in white diet are significantly higher than that of CLD (*P* < 0.01). Takyar and colleagues^30^ analyzed the data from their study comparing LRD with CLD in terms of bowel preparation quality, overall patient tolerance, and side-effect profile by using interim analysis, in which 65 participants with mean age of (56.43 ± 4.80) and (57.25 ± 4.60) between groups were enrolled. This interim analysis indicated LRD has equal effectiveness with CLD and the incidence of side effect profile was not different between these 2 regimes, whereas this study also suggested that LRD did not improve the overall patient tolerance relative to CLD. Although patient tolerance in this study is contrary to our findings, the small sample size in Takyar's study may account for this discrepancy. Tan^31^ and colleagues explored the comparative efficacy of LRD combined with low volume solution compared to standard bowel preparation (CLD) by using the data from 101 adult patients. Same with our findings, this study found LRD not only did not compromise the bowel preparation, but bettered patient tolerance as well. Remaining^32^ was performed by Walter and colleagues used BBPS to assess the bowel preparation quality and supported LRD was not-inferior to CLD, in keeping with our findings.

Moreover, LRD have some advantages for bowel preparation prior to colonoscopy compared to CLD; however, the disadvantages and contraindications of LRD should also be noted. Because the LRD is a diet regime which is designed to decrease the mechanical stimulation of intestinal tract and prolong the intestinal transiting time through reducing the food residue. And thus it can promote wound healing. For these purposes, the diet regime will restrict the food elements which can increase bowel activity. Given this point, long-term use of this diet regime will cause deficiency of vitamin including vitamin C and folic acid and mineral substances such as potassium and calcium. In addition, constipation will occur when 1 is prescribed to long-term use LRD due to sufficient hydrated is not consumed. Certainly, sufficient calorie and protein should also be took in if long-term use of this given diet regime. Considered from these disadvantages, patients with vitamin and protein deficiency and lower intestinal tract mobility, especially patients with lower gastrointestinal motility diseases such as constipation, should not be prescribed to use the LRD regime.

The power, a statistical index, is the probability of correctly rejecting the null hypothesis. That said, alternative hypothesis (H1) is true, so the statistical power is 1 – *β*.^33^ Typically, *α* is set to be as 0.05 so that to guard against Type I error, while β evaluates to 0.20 to guard against Type II error.^34^ As a result, reality just can be maximum determined when power of statistical test is >80%. For all eligible original studies, no more than 3 outcome measures of interest reported by 2 studies^14,25^ have a power of >80%, and remains were all <80%. Most importantly, the powers of 2 outcomes including patient tolerance and compliance with dietary regime reported by Rapier et al^5^ were just 0.1% and 0.3%, respectively. Although the meta-analyses were used to aggregate the sample size for the purpose of increasing the precision of result, the powers of our findings were also unsatisfactory. These findings suggested that there is insufficient evidence to investigate the comparative effects between LRD and CLD on the day before colonoscopy and thus additional studies with high quality and large scale are still warranted.

We performed this systematic review and meta-analysis based on a comprehensive literature search and critical appraisal of original studies. Therefore, more accurate results for most of outcomes of interest were generated from our study. However, some limitations existed in our meta-analysis were needed to discussed. First, these conference abstracts with essential information were not included to compute summary effect estimates and just performed qualitatively analysis, as well as this may impair the power of our findings. Second, language restriction was imposed in our study and more eligible studies could be captured if the search was extended. Selection bias caused by language restriction may reduce the robustness of this meta-analysis. Third, although we prespecified inclusion criteria, the LRD regime varies slightly from 1 study to the others, and this bias may also negatively affect our findings. Finally, the publication bias was not performed owing to an insufficient number of eligible studies on each outcome measure of interest. So power of these pooled results may be impaired if publication bias existed.

## CONCLUSION

Our meta-analysis did not find difference between LRD and CLD in bowel preparation before colonoscopy. Although significant differences were not apparent in efficacy of colon cleansing, compliance with recommended bowel preparation regime and AEs, patient tolerance and willings to repeat the same bowel preparation in future were improved in the LRD group. With the best available evidence, LRD could be recommended to be as a standard regime for bowel preparation prior to colonoscopy.

## Supplementary Material

Supplemental Digital Content

## References

[1] Johnson DA Barkun AN Cohen LB Optimizing adequacy of bowel cleansing for colonoscopy: recommendations from the US multi-society task force on colorectal cancer. *Am J Gastroenterol* 2014; 109:1528–1545. 25223578 10.1038/ajg.2014.272

[2] Enestvedt BK Tofani C Laine LA 4-Liter split-dose polyethylene glycol is superior to other bowel preparations, based on systematic review and meta-analysis. *Clin Gastroenterol H* 2012; 10:1225–1231. 10.1016/j.cgh.2012.08.029 22940741

[3] Quintero E Castells A Bujanda L Colonoscopy versus fecal immunochemical testing in colorectal-cancer screening. *New Engl J Med* 2012; 366:697–706. 22356323 10.1056/NEJMoa1108895

[4] Zauber AG Winawer SJ O’Brien MJ Colonoscopic polypectomy and long-term prevention of colorectal-cancer deaths. *N Engl J Med* 2012; 366:687–696. 22356322 10.1056/NEJMoa1100370 PMC3322371

[5] Rapier R Houston C A prospective study to assess the efficacy and patient tolerance of three bowel preparations for colonoscopy. *Gastroenterol Nurs* 2006; 29:305–308. 16974167 10.1097/00001610-200607000-00007

[6] Harewood GC Sharma VK de Garmo P Impact of colonoscopy preparation quality on detection of suspected colonic neoplasia. *Gastrointest Endosc* 2003; 58:76–79. 12838225 10.1067/mge.2003.294

[7] Froehlich F Wietlisbach V Gonvers JJ Impact of colonic cleansing on quality and diagnostic yield of colonoscopy: the European Panel of Appropriateness of Gastrointestinal Endoscopy European multicenter study. *Gastrointest Endosc* 2005; 61:378–384. 15758907 10.1016/s0016-5107(04)02776-2

[8] Cohen SM Wexner SD Binderow SR Prospective, randomized, endoscopic-blinded trial comparing precolonoscopy bowel cleansing methods. *Dis Colon Rectum* 1994; 37:689–696. 8026236 10.1007/BF02054413

[9] Ness RM Manam R Hoen H Predictors of inadequate bowel preparation for colonoscopy. *Am J Gastroenterol* 2001; 96:1797–1802. 11419832 10.1111/j.1572-0241.2001.03874.x

[10] Mahan KL EscottStump SK, eds. Krause's Food and Nutrition Therapy 12th ed. Pennsylvania: Saunders Elsevier; 2008 Accessed Jun 20, 2015.

[11] Stolpman DR Solem CA Eastlick D A randomized controlled trial comparing a low-residue diet versus clear liquids for colonoscopy preparation: impact on tolerance, procedure time, and adenoma detection rate. *J Clin Gastroenterol* 2014; 48:851–855. 25296243 10.1097/MCG.0000000000000167

[12] Sipe BW Fischer M Baluyut AR A low-residue diet improved patient satisfaction with split-dose oral sulfate solution without impairing colonic preparation. *Gastrointest Endosc* 2013; 77:932–936. 23531424 10.1016/j.gie.2013.01.046

[13] Nelms M Sucher K Long S, eds. Nutrition Therapy and Pathophysiology. California: Thomson; 2007 Accessed Jun 20, 2015.

[14] Delegge M Kaplan R Efficacy of bowel preparation with the use of a prepackaged, low fibre diet with a low sodium, magnesium citrate cathartic vs. a clear liquid diet with a standard sodium phosphate cathartic. *Aliment Pharmacol Ther* 2005; 21:1491–1495. 15948817 10.1111/j.1365-2036.2005.02494.x

[15] Park D Park SH Lee SK Efficacy of prepackaged, low residual test meals with 4L polyethylene glycol versus a clear liquid diet with 4L polyethylene glycol bowel preparation: a randomized trial. *J Gastroen Hepatol* 2009; 24:988–991. 10.1111/j.1440-1746.2009.05860.x 19638081

[16] Nguyen DL Nguyen ET Asombang AW The effect of low-residue diet on quality of bowel preparation on screening colonoscopy: a meta-analysis of randomized control trials. *Gastroenterology* 2015; 148:S600.

[17] Moher D Liberati A Tetzlaff J Preferred reporting items for systematic reviews and meta-analyses: the PRISMA statement. *Ann Intern Med* 2009; 151:264–269.W264. 19622511 10.7326/0003-4819-151-4-200908180-00135

[18] Higgins JP Green S, eds. Cochrane Handbook for Systematic Reviews of Interventions Version 5.1.0. Oxford: The Cochrane Collaboration, 2011 Updated March 2011. www.cochrane-handbook.org Accessed Jun 20, 2015.

[19] Booth AM Wright KE Outhwaite H Centre for Reviews and Dissemination databases: Value, content, and developments. *Int J Technol Assess* 2010; 26:470–472. 10.1017/S0266462310000978 20923587

[20] Zeng X Zhang Y Kwong JS The methodological quality assessment tools for preclinical and clinical studies, systematic review and meta-analysis, and clinical practice guideline: a systematic review. *J Evid Based Med* 2015; 8:2–10. 25594108 10.1111/jebm.12141

[21] He Z Tian H Song A Quality appraisal of clinical practice guidelines on pancreatic cancer: a PRISMA-compliant article. *Medicine (Baltimore)* 2015; 94:e635. 25816030 10.1097/MD.0000000000000635 PMC4554013

[22] Song GM Tian X Shuai T Treatment of adults with treatment-resistant depression: electroconvulsive therapy plus antidepressant or electroconvulsive therapy alone? evidence from an indirect comparison meta-analysis. *Medicine (Baltimore)* 2015; 94:e1052. 26131818 10.1097/MD.0000000000001052 PMC4504538

[23] Tian X Zhou JG Zeng Z Cetuximab in patients with esophageal cancer: a systematic review and meta-analysis of randomized controlled trials. *Med Oncol* 2015; 32:127. 25794489 10.1007/s12032-015-0521-2

[24] Faul F Erdfelder E Buchner A Statistical power analyses using G^∗^Power 3.1: tests for correlation and regression analyses. *Behav Res Methods* 2009; 41:1149–1160. 19897823 10.3758/BRM.41.4.1149

[25] Melicharkova A Flemming J Vanner S A low-residue breakfast improves patient tolerance without impacting quality of low-volume colon cleansing prior to colonoscopy: a randomized trial. *Am J Gastroenterol* 2013; 108:1551–1555. 24091500 10.1038/ajg.2013.21

[26] Scott SR Raymond PL Thompson WO Efficacy and tolerance of sodium phosphates oral solution after diet liberalization. *Gastroenterol Nurs* 2005; 28:133–139. 15832114 10.1097/00001610-200503000-00008

[27] El-Baba MF Padilla M Houston C A prospective study comparing oral sodium phosphate solution to a bowel cleansing preparation with nutrition food package in children. *J Pediatr Gastroenterol Nutr* 2006; 42:174–177. 16456411 10.1097/01.mpg.0000189353.40419.31

[28] Spada C Riccioni ME Hassan C PillCam colon capsule endoscopy: a prospective, randomized trial comparing two regimens of preparation. *J Clin Gastroenterol* 2011; 45:119–124. 20463587 10.1097/MCG.0b013e3181dac04b

[29] Butt J Bunn CE Eldho P The white diet is preferred and better tolerated than a clear fluid diet without hindering successful bowel preparation for colonoscopy. *Gastrointest Endosc* 2014; 79:AB480.

[30] Takyar V Singh N Patel K Low-residue diet in colon preparation prior to screening colonoscopy: a review of literature. *Am J Gastroenterol* 2014; 109:S218–S219.

[31] Tan JYC Nauze RL Bunn CE A single-blind, randomized non inferiority study of the effectiveness and tolerability of low volume bowel preparation in combination with a low residue diet compared to standard bowel preparation prior to morning colonoscopy. *Gastrointest Endosc* 2014; 79 (5 suppl. 1):AB179–AB180.

[32] Walter J Patel A Matro R The impact of diet liberalization on bowel preparation for colonoscopy. *Am J Gastroenterol* 2013; 108:S162. 10.1055/s-0043-101694 PMC5375955 28382323

[33] Muncer S Taylor S Craigie M Power dressing and meta-analysis: incorporating power analysis into meta-analysis. *J Adv Nurs* 2002; 38:274–280. 11972663 10.1046/j.1365-2648.2002.02177.x

[34] Mizuguchi T Kawamoto M Meguro M Laparoscopic hepatectomy: a systematic review, meta-analysis, and power analysis. *Surg Today* 2011; 41:39–47. 21191689 10.1007/s00595-010-4337-6

