# Supplementary material for: Regime for Bowel Preparation in Patients Scheduled to Colonoscopy: Low-Residue Diet or Clear Liquid Diet? Evidence From Systematic Review With Power Analysis
Source: Medicine (Baltimore). 2016 Jan 8;95(1):e2432. doi: 10.1097/MD.0000000000002432 (PMC4706267; doi:10.1097/MD.0000000000002432)
Supplement: Supplemental Digital Content [file medi-95-e2432-s001.pdf]

Recent queries in PubMed

Search	Query	Items found	Time
#10	Search ((((((((colonoscop*) OR Colonoscopic Surgical Procedure*) OR Colonoscopic Surger*)) OR "Colonoscopy"[Mesh])) AND (((((low-residue diet*) OR low residue diet*) OR LRD) OR NutraPrep) OR low residue product*)) AND ((((((clear liquid diet*) OR clear-liquid diet*) OR CLD) OR clear liquid product*) OR clear-liquid product*) OR clear water)) AND (((("Randomized Controlled Trial" [Publication Type]) OR "Randomized Controlled Trials as Topic"[Mesh])) OR random*)) AND ((human) NOT animal)	6	0:50:40
#9	Search (human) NOT animal	12569413	0:49:27
#8	Search ((("Randomized Controlled Trial" [Publication Type]) OR "Randomized Controlled Trials as Topic"[Mesh])) OR random*	971952	0:49:00
#7	Search random*	971702	0:48:39
#6	Search ("Randomized Controlled Trial" [Publication Type]) OR "Randomized Controlled Trials as Topic"[Mesh]	480726	0:48:14
#5	Search (((((clear liquid diet*) OR clear-liquid diet*) OR CLD) OR clear liquid product*) OR clear-liquid product*) OR clear water	15428	0:46:56
#4	Search ((((low-residue diet*) OR low residue diet*) OR LRD) OR NutraPrep) OR low residue product*	614	0:44:33
#3	Search ((((colonoscop*) OR Colonoscopic Surgical Procedure*) OR Colonoscopic Surger*)) OR "Colonoscopy"[Mesh]	32430	0:42:01
#2	Search ((colonoscop*) OR Colonoscopic Surgical Procedure*) OR Colonoscopic Surger*	29479	0:41:47
#1	Search "Colonoscopy"[Mesh]	22526	0:40:51

Embase Session Results (22 Jun 2015)

No.	Query	Results
#25	**#24** AND **'human'**/de	**20**
#24	**#5** AND **#12** AND **#19** AND **#23**	**23**
#23	**#20** OR **#21** OR **#22**	**1135000**
#22	**'randomized controlled trial (topic)'**/exp	**74505**
#21	**'randomized controlled trial'**/exp	**370389**
#20	**random***	**1135000**
#19	**#13** OR **#14** OR **#15** OR **#16** OR **#17** OR **#18**	**22931**
#18	**CLD**	**4068**
#17	**'clear water'**	**17171**
#16	**'clear liquid product*'**	**19**
#15	**'clear-liquid product*'**	**1305**
#14	**'clear liquid diet*'**	**270**
#13	**'clear liquid diet*'**	**817**
#12	**#6** OR **#7** OR **#8** OR **#9** OR **#10** OR **#11**	**9133**
#11	**NutraPrep**	**4**
#10	**LRD**	**683**
#9	**'low residue product*'**	**1298**
#8	**'low-residue product*'**	**7471**
#7	**'low residue diet*'**	**1298**
#6	**'low-residue diet*'**	**326**
#5	**#1** OR **#2** OR **#3** OR **#4**	**56833**
#4	**'colonoscopic surger*'**	**2366**
#3	**'colonoscopic surgical procedure*'**	**363**
#2	**colonoscop***	**56833**
#1	**'colonoscopy'**/exp OR **'colonoscopy'**	**54379**

Recent queries in Science Direct

Search	Results
(((((Colonoscop*)) OR ((Colonoscopic Surgical Procedure*)) OR ((Colonoscopic Surger*))) AND (((low-residue diet*)) OR ((low residue diet*)) OR ((low residue product*)) OR ((low-residue product*)) OR ((NutraPrep)) OR ((LRD))) AND (((clear liquid diet*)) OR ((clear-liquid diet*)) OR ((clear-liquid product*)) OR ((clear liquid product*)) OR ((clear water)) OR ((CLD))) AND (random*) AND (human and not animal) *[All Sources(- All Sciences -)]*	70
(low-residue diet*) OR (low residue diet*) OR (low residue product*) OR (low-residue product*) OR (NutraPrep) OR (LRD) *[All Sources(- All Sciences -)]*	730,391
(Colonoscop*) OR (Colonoscopic Surgical Procedure*) OR (Colonoscopic Surger*) *[All Sources(- All Sciences -)]*	7,232
(Colonoscopic Surger*) *[All Sources(- All Sciences -)]*	74
(Colonoscop*) *[All Sources(- All Sciences -)]*	7,232
(clear liquid diet*) OR (clear-liquid diet*) OR (clear-liquid product*) OR (clear liquid product*) OR (clear water) OR (CLD) *[All Sources(- All Sciences -)]*	505,582
(low residue diet*) *[All Sources(- All Sciences -)]*	772
(Colonoscopic Surgical Procedure*) *[All Sources(- All Sciences -)]*	21
(clear-liquid product*) OR (clear liquid product*) *[All Sources(- All Sciences -)]*	462,838
(clear liquid diet*) OR (clear-liquid diet*) *[All Sources(- All Sciences -)]*	124,595
(low-residue diet*) *[All Sources(- All Sciences -)]*	772
(clear water) OR CLD *[All Sources(- All Sciences -)]*	21,206
human and not animal *[All Sources(- All Sciences -)]*	2,414,198

Search Name: LRD vs. CLD
Last Saved: 22/06/2015 06:02:07.283
Description: **CENTRAL**

ID Search
#1 colonoscop* or Colonoscopic Surgical Procedure* or Colonoscopic Surger* (Word variations have been searched)
#2 MeSH descriptor: [Colonoscopy] explode all trees
#3 #1 or #2
#4 low-residue diet* or low residue diet* or low residue product* or low-residue product* or LRD (Word variations have been searched)
#5 clear liquid diet* or clear-liquid diet* or clear liquid product* or clear-liquid product* or CLD (Word variations have been searched)
#6 MeSH descriptor: [Randomized Controlled Trial] explode all trees
#7 MeSH descriptor: [Randomized Controlled Trials as Topic] explode all trees
#8 random* (Word variations have been searched)
#9 #6 or #7 or #8
#10 human not animal (Word variations have been searched)
#11 #3 and #4 and #5 and #9 and #10

Basic Data Extraction Table Pre-designed

Basic Characteristics of Included into Study					
**Study Information**	**Author**		**Year**		**Country**
**Methods**	**Allocation**				
	**Duration**				
	**Blinding**				
	**Location**				
**Participants**	**Diagnosis**				
	**Age**		**Study Group**		**Control Group**
	**Sex**		**Study Group**		**Control Group**
			Male: Female		Male: Female
	**Length of Illness**		**Study Group**		**Control Group**
			±		±
	**Inclusion criteria**				
	**Exclusion criteria**				
**Interventions**	**Treatment Group**	**Content**			
	**Control group**	**Content**			
**Outcomes**					
**Notes**					
**Drop-Outs**	**Drop out due to**		**Study group**		**Control group**
	**the numbers of patients in the early stage**				
	**the numbers of patients in the late stage**				
**Continuous Data**					
**Outcomes**	**Name of outcome**		**Data Extraction**		
			**Mean**	**SD**	**N**
	**Study group**				
	**Control group**				
**Binary Data**					
**Outcomes**	**Name of Outcome**		**Data Extraction**		
			**Event number**		**Total number**
	**Study group**				
	**Control group**				
**Other Type Data**					
**Outcomes**	**Name of Outcome**		**Data Extraction**		
			**Median**	**Range**	**N**
	**Study group**				
	**Control group**				

Basic Data Extraction Table Pre-designed

Assessing of Risk of Bias Tool		
**Item**	**Description**	**Risk of Bias**
**Sequence Generation**	Describe the method used to generate the allocation sequence in sufficient detail to allow an assessment of whether it should produce comparable groups	Was the allocation sequence adequately
	Comment:	Unclear
**Allocation Concealment**	Describe the method used to conceal the allocation sequence in sufficient detail to determine whether intervention allocations could have been foreseen	Was the allocation adequately concealed?
	Comment:	Unclear
**Blinding of Participants and Personnel**	Describe all measures used, if any, to blind study participants and personnel from knowledge of which intervention a participant received. Provide any information relating to whether the intended blinding was effective	Was knowledge of the allocated intervention adequately prevented during
	Comment:	Unclear
**Blinding of Outcome Assessors**	Describe all measures used, if any, to blind outcome assessors from knowledge of which intervention a participant received. Provide any information relating to whether the intended blinding was effective.	Was knowledge of the allocated intervention adequately
	Comment:	Unclear
**Incomplete Outcome Data**	Describe the completeness of outcome data for each main outcome, including attrition and exclusions from the analysis. State whether attrition and exclusions were reported, the numbers in each intervention group (compared with total randomized participants), reasons for attrition/exclusions where reported, and any reinclusions in analyses performed by the review authors.	Were incomplete outcome data adequately addressed?
	Comment:	Low Risk
**Selective Outcome Reporting**	State how the possibility of selective outcome reporting was examined by the review authors and what was found.	Are reports of the study free of suggestion of
	Comment:	Low Risk
**Other Bias**	State any important concerns about bias not addressed in the other domains in the tool. If particular questions/entries were re-specified in the review protocol, responses should be provided for each question/entry	Was the study apparently free of other problems that could put it at high risk of bias?
	Comment:	Low Risk
